# The Future Midwife: A PRISMA Scoping Review of Education, Transition, and Work Readiness Among Midwifery Students

**DOI:** 10.3390/healthcare14152352

**Published:** 2026-08-02

**Authors:** Danielle Gleeson, Tracey Tulleners, Jessica Elliott, Dianne Stratton-Maher, Liz Ryan, Jillian Mcculloch, Geraldine Roderick, Thenuja Jayasinghe, Joanne Buckley, Jamie-May Newman, Helen Nutter, Jo Southern, Lisa Beccaria, Georgina Sheridan, Tess Trinder, Haiying Wang, Tao Wang, Sita Sharma, Linda Ng, Jing-Yu (Benjamin) Tan, Daniel Terry

**Affiliations:** 1 School of Nursing and Midwifery, University of Southern Queensland, Ipswich, QLD 4305, Australia; danielle.gleeson@unisq.edu.au (D.G.); tracey.tulleners@unisq.edu.au (T.T.); jessica.elliott@unisq.edu.au (J.E.); dianne.stratton-maher@unisq.edu.au (D.S.-M.); joanne.buckley@unisq.edu.au (J.B.); jo.southern@unisq.edu.au (J.S.); georgina.sheridan@unisq.edu.au (G.S.); emily.wang@unisq.edu.au (H.W.); alison.wang@ecu.edu.au (T.W.); sita.sharma@unisq.edu.au (S.S.); linda.ng@nd.edu.au (L.N.); benjamin.tan@nd.edu.au (J.-Y.T.); 2 Institute for Health, University of Southern Queensland, Springfield, QLD 4300, Australia; liz.ryan@unisq.edu.au (L.R.); jillian.mcculloch@unisq.edu.au (J.M.); geraldine.roderick@unisq.edu.au (G.R.); lisa.beccaria@unisq.edu.au (L.B.); 3 School of Nursing and Midwifery, University of Southern Queensland, Toowoomba, QLD 4350, Australia; thenuja.jayasinghe@unisq.edu.au (T.J.); jamie-may.newman@unisq.edu.au (J.-M.N.); helen.nutter@unisq.edu.au (H.N.); tess.trinder@unisq.edu.au (T.T.); 4 School of Nursing and Midwifery, Edith Cowan University, Joondalup, WA 6027, Australia; 5 School of Nursing and Midwifery, The University of Notre Dame Australia, Fremantle, WA 6160, Australia; 6 Institute of Health and Wellbeing, Federation University Australia, Ballarat, VIC 3350, Australia; 7 School of Nursing, Shiga University of Medical Science, Seta Tsukinowa-cho, Otsu 520-2192, Japan

**Keywords:** midwifery education, student preparedness, clinical practice, transition to practice, competency-based education, educational framework

## Abstract

Background: Preparing midwifery graduates to be confident, competent, and resilient is essential for workforce sustainability, yet students report a disconnect between academic preparation and clinical realities. Increasing care complexity, workforce shortages, and variable clinical learning environments globally highlight the need to examine how educational strategies support effective transition to practice. Objective: To map and synthesise the evidence regarding educational approaches, learning experiences, and transition-to-practice factors that influence preparedness for professional practice among midwifery students, and to identify gaps in the existing literature to inform a coherent and future-ready educational framework. Methods: A PRISMA-ScR-guided scoping review was conducted across seven databases. Data were extracted through a standardised template and analysed using narrative synthesis. Qualitative, quantitative, and mixed-method studies were examined for learning approaches, practice conditions, and transition experiences. Results: Three overarching domains and seven sub-domains were identified. Reflective practice, blended learning, simulation, and collaborative approaches were widely valued but inconsistently implemented. Students frequently experienced transition shock, limited scaffolding of higher-order thinking, variable supervision, with misalignment between curricula, competency frameworks, and clinical expectations, leading to uneven confidence and skill development. Educator capacity constraints particularly in feedback, reflection, and simulation pedagogy were recurrent barriers. Positive clinical environments and structured reflective opportunities consistently strengthened competence, identity formation, and readiness. Conclusion: Midwifery education is evolving towards contemporary, competency-based pedagogies, yet persistent inconsistencies undermine the reliability of graduate preparation. Strengthening curriculum coherence, standardising key learning experiences, investing in educator capability, and embedding structured reflection and simulation are essential. A unified educational framework that integrates these components is required to enable confident, adaptable, and practice-ready midwives.

## 1. Introduction

The transition from student to practicing midwife represents a critical phase in the development of a safe, competent, and resilient midwifery workforce [1,2,3]. Contemporary maternity care is characterised by increasing complexity, rising service demands, workforce pressures, and ongoing changes to models of care [4]. This requires graduates to demonstrate clinical competence, emotional resilience, professional identity, and sound decision-making [5]. Despite completing accredited educational programs, midwifery students and new graduates report feeling underprepared for the practical, relational, and emotional realities of professional practice [1,3]. This gap between educational preparation and workplace expectations has raised concerns among educators, industry partners, and clinical leaders regarding the adequacy of current educational and transition-to-practice models [6].

Preparedness for midwifery practice extends beyond the attainment of technical skills to include critical thinking, communication, autonomy, adaptability, reflective practice, and the ability to work effectively within multidisciplinary teams [7]. Equally important are confidence, resilience, and the development of a strong professional identity, which underpin safe, woman-centred care in unpredictable and emotionally demanding environments [8]. However, existing research highlights substantial variations in how these capabilities are taught, practiced, assessed, and supported across institutions and clinical settings [9]. In addition, differences in models of maternity care, regulatory frameworks, scopes of professional practice, workforce structures, and service delivery contexts can shape the competencies expected of midwives and the educational approaches used to develop them. Consequently, while many educational challenges may be shared internationally, the specific knowledge, skills, and professional capabilities required of midwives may vary across healthcare systems and jurisdictions [4]. Further, fragmented clinical placement structures, inconsistent mentorship, variability in preceptor expertise, educator capacity limitations, and differences in learning environments contribute to variable educational experiences and inconsistencies in graduate readiness [2,3]. These challenges highlight the urgent need to examine how current educational approaches prepare students for contemporary midwifery practice [7].

In response to these concerns, midwifery education has increasingly incorporated blended learning, simulation-based training, structured reflection, and competency-based curricula [10,11]. While these methods show promise, their uptake and implementation remain inconsistent. Simulation varies between institutions, reflective practice is often insufficiently scaffolded, and mentorship models lack standardisation [9,10]. Furthermore, misalignment between academic curricula and clinical expectations continues to create gaps in students’ procedural confidence and decision-making capabilities, particularly during early placements [7]. Students frequently experience transition shock, challenges in applying theoretical knowledge in real-world contexts, and uncertainty navigating hierarchical or inconsistent clinical environments [1,2]. This highlights the importance of understanding the collective impact of educational strategies, clinical learning conditions, and transition supports on student preparedness [3].

Complicating this picture is the broader workforce context. High attrition rates among early career midwives are associated with limited transition support, workload pressures, and emotional strain [4,8]. Ensuring new graduates enter the workforce with confidence, competence, and resilience is therefore vital to sustaining the midwifery profession and ensuring high quality midwifery care. Despite the breadth of existing research, a greater understanding of how education effectively prepares midwifery students for the realities of professional practice is lacking [7]. Most midwifery education studies are fragmented, encompassing elements of curriculum design, teaching strategies, simulation-based learning, and mentorship approaches, which limits the capacity to design effective interventions that bridge the academic-practice divide [3]. Most studies examine isolated components or curriculum design without providing a cohesive framework that reflects the complexity of real-world readiness [6]. Therefore, a comprehensive scoping review is warranted to map the current landscape, identify critical gaps, and inform future educational strategies [2]. A scoping review methodology was chosen because the literature encompasses diverse study designs, educational approaches, and contextual factors, and the objective was to map the breadth of evidence and identify knowledge gaps rather than evaluate intervention effectiveness.

Guided by the Population–Concept–Context (PCC) framework, the review question is: What evidence exists regarding educational approaches, learning experiences, and transition-to-practice factors that influence preparedness for professional practice among midwifery students across educational and clinical contexts?

1. Within this context, the aim of this scoping review was to map and synthesise the evidence regarding educational approaches, learning experiences, and transition-to-practice factors that influence preparedness for professional practice among midwifery students, and to identify gaps in the existing literature. The review therefore seeks to explore how current educational approaches support, or fall short of supporting, students’ readiness to enter the healthcare workforce. The objectives were to: To identify key gaps associated with midwifery student preparedness for professional practice.
2. To explore and scope the key research gaps within midwifery education and transition to the practice literature.
3. To uncover areas where midwifery educational and transition processes may be improved to support student development to enter the workforce.

The insights generated will inform the development of a comprehensive educational framework and targeted transition supports to better prepare midwifery graduates for the complexities of contemporary maternity care.

## 2. Materials and Methods

A scoping review was undertaken to address the aim, and narrative synthesis was implemented as guided by Popay et al. [12], through an iterative, multi-step process. Each identified study was read multiple times and coded using a standardised template to identify key statements and concepts. These were grouped into thematic clusters aligned with the review aims. Aggregation was applied to findings conveying shared meaning. Configuration was used for thematically distinct or conflicting findings. The objectives, analysis methods, inclusion and exclusion criteria were developed and documented, following the PRISMA for Scoping Reviews (PRISMA-ScR) checklist [13], to ensure accurate and complete reporting of findings (see Supplementary File S1). A review protocol was developed a priori and registered with the Open Science Framework (https://osf.io/srcen, accessed on 12 June 2026). The review was conducted in accordance with the protocol.

### 2.1. Search Strategy

A comprehensive literature search was conducted on 12 December 2024, and an updated search was completed on 31 January 2026 using APA PsycINFO, CINAHL, Informit, ProQuest Central, PubMed, Scopus, and Web of Science to identify peer-reviewed literature published between 1 January 2004 and 31 January 2026 examining midwifery student learning and preparedness. The databases were searched using title, keyword, or abstract as supported by individual database functionalities. Within Scopus, searches were restricted to title and abstract fields as preliminary full-text searches retrieved a substantial volume of unrelated records, reducing search precision and screening efficiency. Although this approach may have excluded some potentially relevant studies that did not explicitly reference the search concepts within the title or abstract, this risk was minimised using multiple databases, supplementary hand-searching, and reference list screening of included studies. Key search terms used were organised into concept groups and combined using Boolean operators. Word variations and suffixes were included (Table 1) (see Appendix A). In addition, hand searching and reviewing of reference lists of final included studies was undertaken to uncover any additional studies that may have not been captured by initial searches.

### 2.2. Inclusion and Exclusion Criteria

The review considered articles focused on midwifery students enrolled in undergraduate or postgraduate programs within universities, colleges, or other tertiary institutions. Eligible empirical studies used qualitative, quantitative, or mixed methods designs. Studies were excluded if they did not address midwifery students, early career practitioners, or issues related to educational preparedness. Reviews synthesising primary research (such as systematic, scoping, integrative, or narrative reviews) were also excluded, along with grey literature, to ensure methodological consistency and maintain a focus on peer-reviewed empirical research. In addition, full-text publications in languages other than English were omitted due to concerns about translation accuracy.

### 2.3. Study Screening

All retrieved articles were imported into EndNote (version 21) for management, after duplicate removal occurred, the remaining articles were uploaded onto JBI SUMARI for screening and data extraction. Two reviewers (JM and JE) conducted the initial screening of titles, keywords, and abstracts to exclude irrelevant studies. Full-text screening was then performed independently by two reviewers (JM and DSM), applying the predefined inclusion and exclusion criteria. Each paper was categorised as ‘include,’ ‘exclude,’ or ‘uncertain.’ Any disagreements were resolved through discussion with a third reviewer (TJ for title and abstract, and TT for full-text review) until consensus was reached. Consistent with PRISMA-ScR, no formal quality appraisal was conducted [13]. Scoping reviews are designed to comprehensively map the breadth, scope, and characteristics of available evidence rather than evaluate methodological rigour or exclude studies based on quality. This approach ensures inclusivity across diverse study designs and contexts, which is essential for identifying research gaps and informing future systematic reviews [13,14].

### 2.4. Data Extraction and Analysis

Given the breadth and heterogeneity of the included studies, articles were grouped into thematic clusters aligned with the review objectives. These clusters were allocated to review teams who conducted data extraction and analysis using a standardised extraction template and written instructions. Extracted data were independently checked within each team, with discrepancies resolved through discussion and consultation with a lead researcher (DT) where required. Consensus processes were used throughout the analysis to support consistency in thematic interpretation across review teams.

Oversight of the extraction and analysis process was maintained by a lead researcher (DT), who conducted consistency checks, verified thematic coding against source data, and ensured adherence to the PRISMA-ScR protocol. Following completion of the thematic analyses, all extracted data were collated and synthesised to identify patterns and relationships across domains, ensuring an integrated interpretation of findings.

Textual data were analysed using the narrative synthesis framework described by Popay et al. [12]. Papers were reviewed iteratively to identify key concepts, patterns, and themes. Quantitative studies also contributed textual data, as methodological and outcome heterogeneity precluded meta-analysis. Extracted findings were initially coded according to recurring concepts related to student preparedness, learning approaches, practical learning experiences, and transition to practice. Related codes were grouped into preliminary thematic categories and refined through iterative comparison across studies.

Initial findings were organised into five preliminary domains and associated sub-domains reflecting the review aims. Through iterative synthesis, conceptually related findings were aggregated to strengthen interpretations, while distinct findings were configured to expand and refine understanding of the topic [12]. Through this process, the preliminary domains were consolidated into seven interconnected sub-domains, which were subsequently organised into three overarching domains [12].

## 3. Results

**Figure 1:**
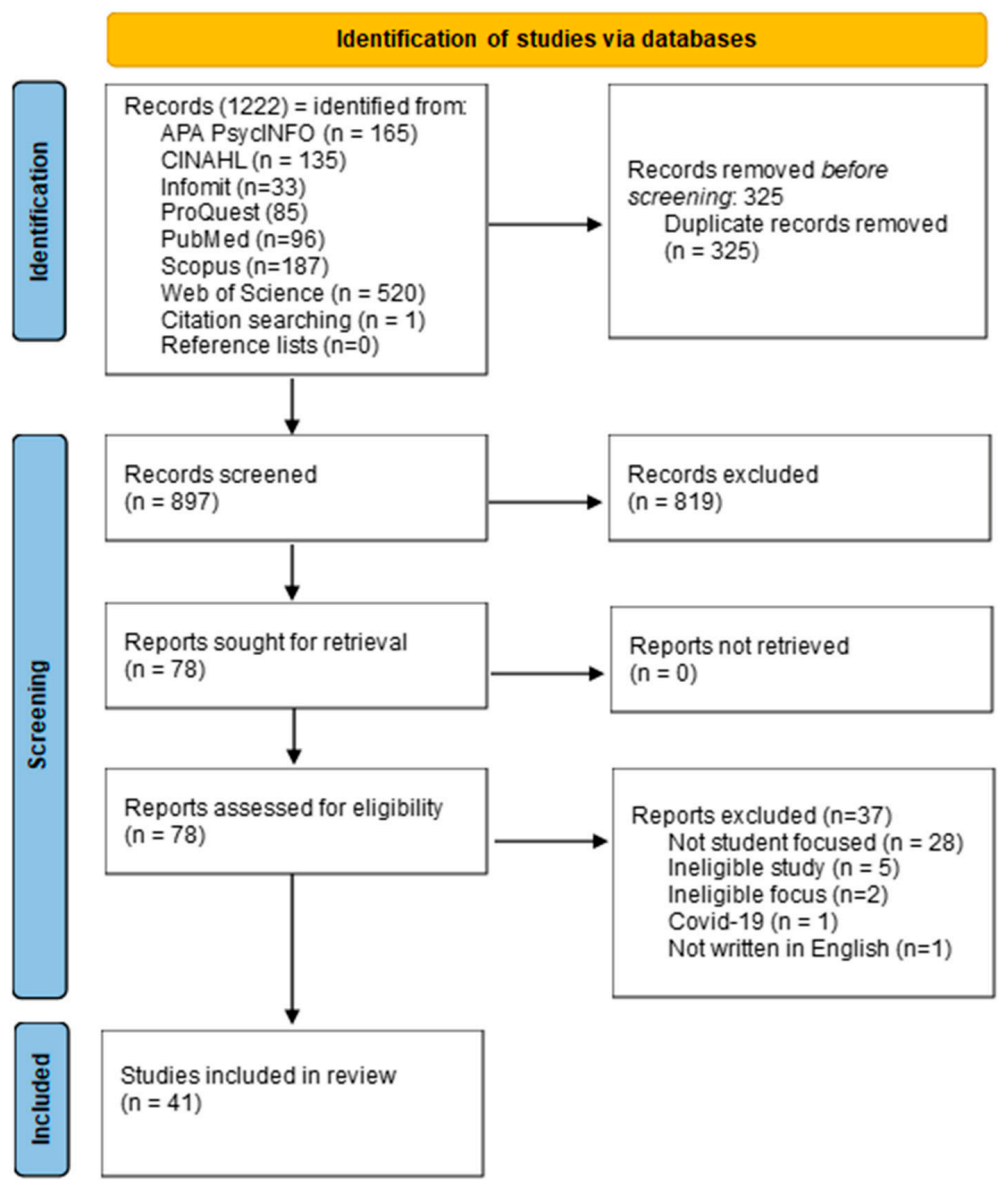
PRISMA-ScR flow diagram.

The literature search yielded 1222 potentially relevant publications and after removing duplicates (*n* = 325), including those that did not meet the inclusion criteria (*n* = 855). A total of 41 studies were agreed upon for inclusion in the literature review (Figure 1).

The final group of publications included qualitative studies (*n* = 18), which represented the largest proportion of the literature. This was followed by mixed methods studies (*n* = 6), cross-sectional studies (*n* = 3), descriptive study or report (*n* = 3), evaluation studies (*n* = 2), quasi-experimental studies (*n* = 2), and action research (*n* = 2). In addition, there were also a case study (*n* = 1), a conceptual paper (*n* = 1), discussion paper (*n* = 1), ethnography (*n* = 1), and a reflective practice paper (*n* = 1). Specifically, they were conducted across multiple countries, including Australia (*n* = 12), UK (*n* = 11), Iran (*n* = 3), Ireland (*n* = 3), United States (*n* = 1), New Zealand (*n* = 1), Sweden (*n* = 1), Belgium (*n* = 1), Italy (*n* = 1), Norway (*n* = 1), South Africa (*n* = 1), Kenya (*n* = 1), China (*n* = 1), Netherlands (*n* = 1), and Ethiopia (*n* = 1), while one study was an international multi-country study (*n* = 1) (Appendix B).

**Figure 2:**
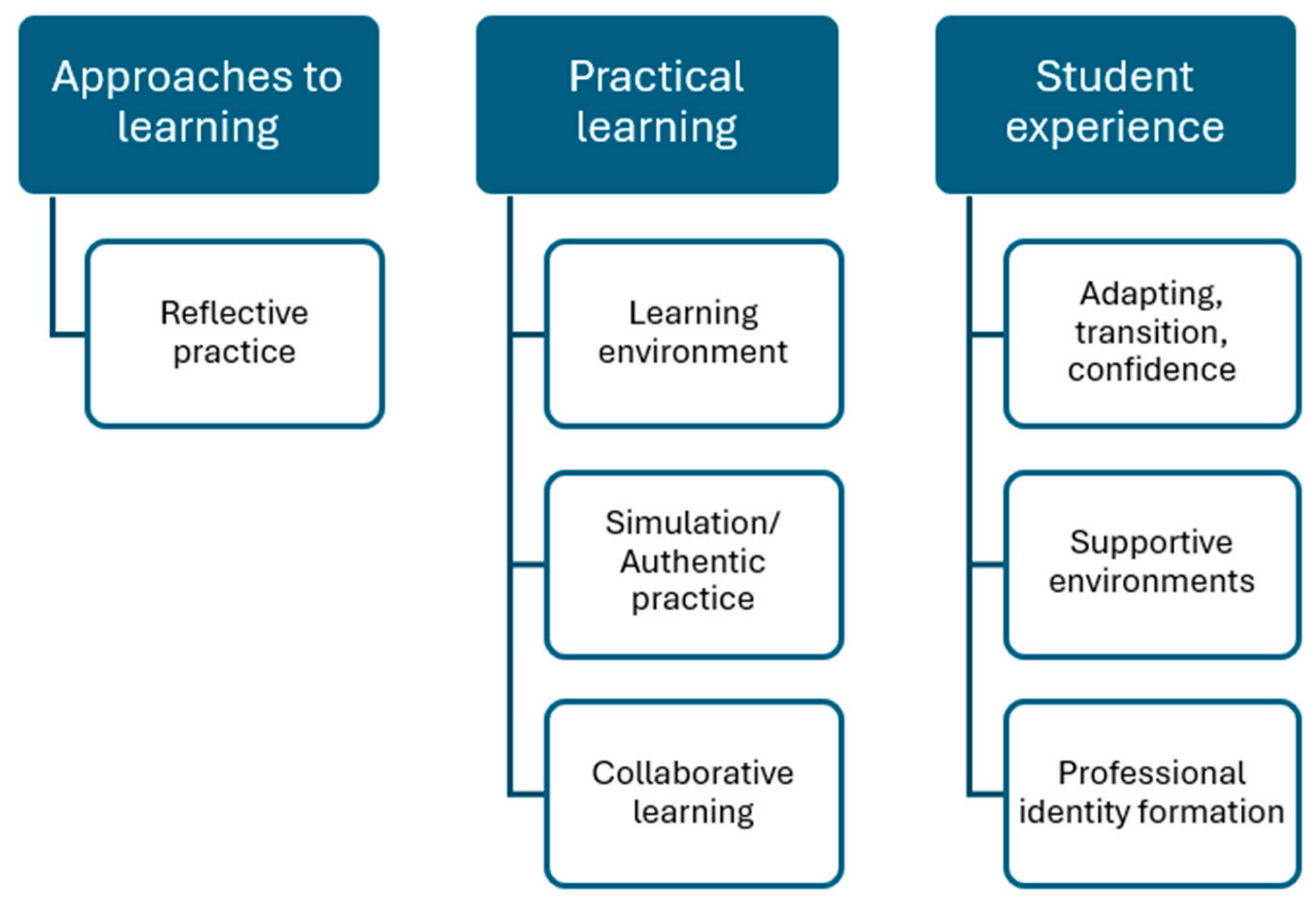
Identified domains and sub-domains.

The narrative synthesis framework enabled the findings to be synthesised and integrated across domains by identifying recurring patterns and relationships rather than isolated narratives. Three overarching domains, informed by the research objectives, included Approaches to learning, Practical learning, and Student experience, along with seven sub-domains (Figure 2). Each domain and sub-domain was informed by findings reported across multiple included studies, with representative studies cited throughout the narrative synthesis.

### 3.1. Approaches to Learning

The literature demonstrates an evolution in midwifery education from traditional, content-heavy instruction toward student-centred approaches that integrate feedback, competence, and professional identity formation [15,16]. Educational design throughout is consistently framed as a dynamic process aimed at preparing students for complex, practice-based environments rather than simply transmitting knowledge [15,16].

Technology-enhanced and blended learning approaches were prominent throughout the literature, with digital platforms and interactive online environments associated with improved accessibility, flexibility, and engagement of students [17,18,19]. These strategies supported autonomy and adaptability, enabling students to manage learning across diverse contexts. Despite these benefits, challenges that persisted were associated with equitable access to technology and continued perceptions that online learning lacks the value of face-to-face delivery [17,18].

Competency-based and practice-oriented learning approaches were strongly represented, reflecting the imperative to align curricula with professional standards and clinical performance expectations [20,21,22]. However, several articles indicated gaps in readiness for practice, with students in certain locations failing to meet essential competencies for safe care [21], suggesting the need for structured supervision, authentic assessment, and targeted support strategies to ensure competence and confidence [21].

Further, feedback-driven learning was consistently identified as a cornerstone of effective education [23,24]. Clear, documented feedback enhanced self-assessment accuracy and was more positively viewed among students than verbal feedback [23]. In addition, sustained educator engagement was associated with improved motivation and professional development, reinforcing the formative role of feedback in shaping competence [17].

The development of critical thinking and clinical reasoning was also recognised within the literature as essential, but often underemphasised. Studies indicated critical thinking was assumed to develop incidentally through experience rather than through explicit instruction [25]. Several studies highlighted the value of structured reasoning processes in supporting safe and adaptive practice [25]. Nevertheless, it was also demonstrated that inconsistent supervision, limited clinical exposure, and variable institutional standards were linked to competence gaps, emphasising the need for integrated theoretical, and practical learning supported by ongoing evaluation and redesign [20,22].

#### Reflective Practice

Building on the approaches to learning, reflective practice emerged as a prominent theme within the literature, with studies highlighting its role in addressing gaps in student preparedness and supporting early career development [26,27,28]. Reflection was consistently described as a transformative process that fosters resilience, critical thinking, and professional competence [29,30]. However, despite its prominence in midwifery curricula, implementation was identified as being often fragmented and insufficiently scaffolded, leaving students struggling to engage meaningfully, particularly in the early stages of their learning [26,27].

This challenge is compounded by inconsistent mentorship and limited feedback, which hindered the development of self-awareness and autonomous decision-making [28]. Reflection also played a critical role in mitigating emotional and cognitive challenges during the transition from student to midwife. Bradshaw, Murphy Tighe [29] reported that structured and informal reflective sessions assisted students manage stress during their training, process complex experiences, and strengthen professional identity. Yet, time allocated for reflection was often undervalued in clinical settings, with some health professionals dismissing it as a ‘break’ rather than an essential component of education [29].

Pedagogical strategies such as storytelling and legitimate peripheral participation, where situated learning enables newcomers to become experts by engaging in and gradually moving from the periphery to the core of a community of practice [31], were highlighted as powerful tools for reflective learning [32,33]. Dialogue with experienced midwives and narrative sharing enabled students to internalise tacit knowledge, clarify emotional responses, and connect theory to practice. Written reflection further supported cognitive and emotional development, allowing students to move from fragmented observations to holistic evaluations and articulate evolving professional perspectives [30].

Despite these benefits, systemic challenges persist. For example, variability in preceptor engagement and feedback was shown to undermine the effectiveness of reflective learning, and the absence of standardised approaches also risked reducing its value [28,29]. Several studies highlighted the importance of structured reflection, mentorship, and institutional support. As such, reflection, through storytelling, writing, and experiential dialogue, was commonly associated with the development of competence and resilience among midwifery students [27,33].

### 3.2. Practical Learning

In addition to the approaches to learning, practical learning is the cornerstone of midwifery education, providing the context where theoretical knowledge is transformed into practical competence. Four interrelated sub-themes shaping this process were identified, including the learning environment and educator capacity, simulation as a pedagogical tool, collaborative learning approaches, and exposure to authentic midwifery practice. Together, these sub-themes are discussed to highlight the complexity of practical learning.

#### 3.2.1. Learning Environment and Educator Capacity

The quality of the learning environment plays a pivotal role in shaping the success of a midwifery student’s educational journey. The capacity of the environment determines the breadth of learning opportunities available [34], yet limitations of equipment and technological resources can undermine effective skill development [35,36]. Simulated environments offer a valuable solution, providing safe and structured spaces that support cognitive growth and facilitate the transfer of knowledge and skills into practice [26,37].

Equally important is the expertise and preparedness of educators. Collaborative teaching models that bridge academic and clinical domains were identified as essential for aligning theoretical knowledge with practical application [38]. However, gaps in educator preparedness particularly in teaching advanced clinical skills were noted as a barrier to student development [39]. These deficiencies in teaching proficiency, coupled with workload pressures and inconsistent expectations between students and preceptors, were shown to hinder learning during placements [32,34,36,40]. To address these challenges, studies indicated that educators require access to current resources and opportunities for ongoing professional development to maintain currency and deliver high-quality midwifery education [39].

#### 3.2.2. Simulation and Authentic Practice

Building on the learning environment, structured approaches such as simulation have emerged as a vital complement to traditional teaching, offering safe and controlled environments where students can develop essential skills and decision-making capabilities before entering real-world practice [32,35,37]. Simulation plays a pivotal role in developing critical thinking among midwifery students by providing a safe environment to apply actions, make decisions, and engage in reflective analysis [41]. It offers exposure to complex and varied clinical scenarios, enabling students to test different approaches to care and fosters self-awareness and self-evaluation [42]. Beyond technical skills, simulation was shown to promote deeper understanding of the contextual and cultural factors that shape midwifery practice and clinical decision-making [32,35,37].

The effectiveness of simulation is enhanced when cognitive and analytical thinking are purposefully integrated into activities, ensuring that reflective decision-making is explicit and intentional [26]. Structured opportunities for feedback and dialogue with educators further strengthen students’ ability to justify their actions and refine clinical reasoning, reinforcing both reflective and critical thinking skills [36,39,43]. Overall, the acquisition of clinical skills and confidence was a consistent finding across the identified studies. Simulation-based learning provided safe, structured opportunities for students to practise procedures, test decisions, and apply theoretical knowledge in realistic contexts [41,42]. Repeated exposure to simulation enhanced competence, supported reflective learning, and built self-confidence. These experiences also facilitated adaptability and independent decision-making as students transitioned into real-world midwifery practice [37,43].

As such, it was evident that exposure to authentic midwifery practice and midwifery-led models of care significantly enhance student preparedness for practice. Authentic experiences fostered the development of autonomy, advocacy, and clinical intuition among students, which were shown to be key attributes for early career success [16,32,40]. The quality of the authentic learning environment emerged as a pivotal factor in shaping student experiences. Supportive professional placements where students felt welcomed and respected were associated with enhanced learning, skill acquisition, and professional identity formation [32,44]. Conversely, hostile or unsupportive environments characterised by ageism, discrimination, bullying, and poor clinical conditions eroded student confidence and compromised their development [15,45,46]. Inconsistent supervision and the absence of structured transition protocols further hindered students’ ability to integrate into clinical teams and develop clinical intuition [47].

#### 3.2.3. Collaborative Learning

In addition to simulation, collaborative learning emerged as a significant sub-theme in the literature. Students reported benefits from learning alongside peers and receiving support from preceptors, which enhanced confidence and skill development [32,42,48]. Practising skills in a safe, peer-supported environment prior to professional placement was particularly valued, as it fostered reciprocity and continuity in teaching and learning when transitioning to real-world settings [36,48]. Cross-institutional collaboration was also identified as an important enabler of student learning. Partnerships between facilities help maintain program integrity and ensure shared expectations, supporting consistency in educational experiences [35,40]. Such collaboration promotes understanding of interdependence and relational dynamics within workplace culture, reinforcing the collective responsibility for high-quality clinical education.

### 3.3. Student Experience

Beyond formal learning approaches and clinical skill development, student experiences form a critical dimension of midwifery education, reflecting the progression from early uncertainty to professional identity. Three interrelated sub-themes that inform student experience include adapting, transition and confidence, supportive learning environments, and professional identity formation.

#### 3.3.1. Adaptation, Transition, and Confidence

Students consistently reported difficulties in adapting to the academic and clinical demands of midwifery education. The transition to university was marked by challenges in balancing study, professional placement, and personal responsibilities, often compounded by insufficient institutional support [15,29]. A lack of clarity around academic expectations, assessment criteria, and clinical competencies further exacerbated feelings of uncertainty and unpreparedness for learning and professional development [45,49].

Students’ practical experiences were often marked by transition shock and difficulty applying classroom knowledge in unfamiliar clinical environments [34,50]. Limited hands-on practice and misalignment between curriculum and professional placement expectations contributed to stress and confusion, with students expressing a need for more realistic preparation [34,44]. For example, students demonstrated technical proficiency; however, a significant proportion lacked confidence in executing essential clinical procedures for real world readiness [51]. Skills such as episiotomy, catheterisation, medication administration, and the management of obstetric emergencies were identified as areas of concern [38,39,40]. Overall, Amod and Mkhize [52] reported students feeling incompetent in performing core procedures.

Moreover, decision-making capacity was often underdeveloped, particularly in situations where students encountered conflicting clinical practices or hierarchical dynamics between health professionals that undermined their autonomy [37]. Nevertheless, as learning progressed, integrative strategies such as reflection and observation of preceptors helped link theory to practice, while reinforcing professional conduct [32,53]. However, inconsistent supervision and continuity across health care sites remained barriers to skill development [29,30].

#### 3.3.2. Supportive Environments

Supportive environments were shown to be critical in assisting student transition into practice. Preceptor and mentor guidance fostered confidence, while peer relationships enhanced engagement and early identity formation [32,44,50]. Peer mentorship during clinical placements provided a valuable buffer against inconsistent preceptorship, offering emotional support and practical guidance [44,47]. These findings highlight the importance of cultivating a culture of support and collaboration within both educational and clinical settings. In this sense, a culture of inclusion and psychological safety was associated with motivation and learning [15]. As students advanced, structured supervision and consistent feedback became essential for skill mastery and professional growth [30]. Conversely, discriminatory or unsupportive clinical cultures undermined morale and learning, highlighting the need for inclusive and responsive environments [46].

#### 3.3.3. Professional Identity Formation

The development of professional identity began early, with students often struggling with role transition during initial professional placements [45]. Reflective activities and storytelling supported emotional processing and identity formation [33]. Continuity of care and early exposure fostered belonging and purpose [15,45,53]. Later stages were characterised by increasing autonomy and emotional maturity, as students progressed from dependence to independent decision-making [30]. Final placements consolidated this growth, affirming readiness for practice and reinforcing professional identity [29,40].

Nevertheless, students encountered structural and curriculum-related barriers from the beginning of their training. In undergraduate programs, first year students faced limited hands-on practice, faculty shortages and inadequate simulation resources [34,39]. These barriers contributed to early stress and hindered skill acquisition [34,39,50]. Students also reported curricular and structural inconsistencies across sites, which impacted skill consolidation and readiness for practice [29,46]. As students advanced, they called for curriculum redesign, standardisation of clinical procedures and greater involvement in educational planning to ensure development alignment and competency progression [54]. Students frequently advocated for curriculum redesign, standardisation of clinical procedures, and greater involvement in educational planning.

## 4. Discussion

This scoping review sought to identify gaps in the educational preparedness and transition to practice of midwifery students for their early career roles. Student experiences revealed considerable variation in how midwifery programs structure learning, support skill and identity development, and prepare students for clinical practice [7,9]. Studies were mapped across the three domains and resulted in seven interconnected approaches to learning. These approaches to learning included reflective and self-directed learning, blended and technology-enhanced learning, competency-based and practice-oriented learning, feedback-driven and relational learning, critical thinking and clinical reasoning approaches, self-efficacy and identity-focused learning, and curriculum alignment and quality enhancement.

Despite a range of approaches to learning identified in the various studies, this review found that higher-order skills such as critical thinking, clinical reasoning and professional judgement are often assumed rather than intentionally and systematically taught [55,56]. Reflective practice is frequently required but rarely scaffolded; students are often ‘told to reflect’ without clear models, frameworks, criteria, or feedback, and it is even viewed by some supervisors as a ‘break’ rather than an essential aspect of practice [10,29]. Reflective practice was consistently presented as central to resilience, critical thinking, clinical reasoning, professional identity and professional judgement, yet variability in preceptor engagement, including their own education, preparedness, and self-efficacy for these roles, along with their capacity to provide feedback undermines its effectiveness [9,57].

Students value hands-on experience, simulation, peer learning, and positive clinical environments that foster safety, participation, and constructive feedback; however, access to simulation, blended learning and collaborative activities varies across programs [7,58]. Simulation, blended learning and peer learning were often reported as isolated initiatives driven by local champions, with limited evidence of program-wide curriculum mapping to ensure coherent sequences of experiential learning [7,59]. Likewise, continuity of care experiences were core to midwifery education in some locations but inconsistently offered in others [7,59]. This fragmentation also reflects uneven integration, resulting in pockets of excellence surrounded by systemic inconsistency.

There is limited attention to resilience, autonomy, and collaboration as explicit educational outcomes, despite evidence that professional identity formation and motivation depend on transformative educational approaches that foster judgement, critical self-evaluation, and accountability [5,8]. Although midwifery education has included many elements of contemporary, competency-based pedagogy; inconsistent implementation, limited standardisation and constrained educator capacity continue to undermine its potential. This suggests that strengthening coherence across the identified educational approaches may offer a pathway toward more consistent and reliable workforce preparation.

The review extends the current evidence base in several important ways. Previous reviews have primarily focused on either the transition experiences of newly qualified midwives or broader curriculum developments within midwifery education [6,7]. In contrast, this review synthesised evidence across educational approaches, practical learning experiences, and student transition experiences simultaneously, enabling the identification of three overarching domains and seven interconnected sub-domains influencing preparedness for practice. By integrating these previously fragmented areas of inquiry, the review advances understanding beyond individual educational interventions and provides a conceptual framework that illustrates how pedagogical approaches, clinical learning experiences, and developmental student outcomes collectively contribute to work readiness. Importantly, the review moves beyond describing educational strategies to conceptualising how the relationships between educational, clinical, and developmental factors influence preparedness for practice. This synthesis offers a more comprehensive foundation for future educational planning, curriculum development, and empirical testing than has previously been available.

An important consideration arising from this review is the influence of healthcare system contexts on midwifery education and preparedness for practice. The included studies were conducted across countries characterised by different models of maternity care, regulatory requirements, scopes of practice, and workforce structures [4,7,60]. These contextual differences inevitably influence educational priorities, curriculum design, clinical learning opportunities, and expectations of graduate competence [4,60]. Therefore, while common educational themes emerged across the literature, their implementation and significance should be interpreted within the healthcare systems in which they are situated.

At the same time, the findings suggest that several educational challenges transcend national boundaries, including transition shock, inconsistent supervision, educator capacity constraints, professional identity development, and the need for structured reflective and simulation-based learning [29,34,39,41,50]. The inclusion of studies from diverse healthcare and educational contexts enabled the identification of these shared issues and highlights opportunities for reciprocal international learning [7]. Educational innovations and lessons arising from different healthcare and educational contexts may offer valuable insights across jurisdictions, irrespective of resource setting, model of care, or regulatory environment [4,7,60]. This aligns with the International Confederation of Midwives view that core quality indicators for midwifery education can assist midwives globally to meet the ICM international definition of the midwife and contribute to systematic improvements within midwifery education programs internationally [60].

While many studies described a shift toward more student-centred, competency-based, and reflective approaches, implementation remained uneven and often dependent on local context, educator capacity, and placement environments [7]. The findings also reflected a gradual shift from traditional, content-heavy models towards more student-centred, competency-based education; however, this shift is often inconsistently implemented and under-theorised [55]. Additionally, there is a lack of standardisation and weak alignment with competency frameworks. Although midwifery standards of practice are well established, their operationalisation in curricula and assessment is often unclear [4,7].

Educator and supervisor capacity constraints further undermine implementation, with practice education facilitators and clinical mentors reporting high workloads and inconsistent expectations between midwifery students and preceptors [32,34,36,40]. When educators are under-resourced, feedback becomes inconsistent, reflective activities are not adequately supported, and innovative approaches such as simulation are difficult to sustain [30]. Many studies [15,29,30,34,37,46,50] also described transition shock, emotional burden, and a perceived gap between the ‘university midwife’ and the ‘real-world midwife’, with supportive supervision and clear feedback unevenly available.

### 4.1. Implications for Education and Research

The findings of this review demonstrate that, although midwifery education incorporates a wide range of pedagogical, reflective, and clinical strategies, the evidence base remains fragmented and inconsistently applied across programs and practice settings [7,59]. The educational implications presented represent the synthesis and interpretation of recurring themes identified across the included studies and are intended to highlight potential directions for educational development and future research. Student preparedness is shaped not by isolated interventions but by the interaction of learning approaches, educator capability, simulation quality, reflective practice, clinical learning environments, and transition supports; yet these components are rarely implemented as an integrated whole [9]. This fragmentation limits the sector’s capacity to reliably prepare graduates for contemporary practice and underscores the need for a coherent, evidence-informed educational framework that aligns academic preparation with clinical expectations, strengthens reflective and simulation-based learning, enhances educator capability, and ensures supportive, high-quality clinical environments [4]. Addressing these gaps may contribute to improved graduate confidence, preparedness, and transition experiences; however, the effectiveness of specific educational approaches was not evaluated within this review [1,2].

Collectively, the review suggests curricula may benefit from explicit alignment with competencies encompassing knowledge, skills, professional identity, resilience, and collaboration, consistent with contemporary midwifery capability frameworks [4]. The interpretation emerged across studies highlighting curriculum misalignment, competence gaps, and variability in educational experiences [7,20,21,40]. Simulation, continuity-of-care experiences, and structured reflection were consistently identified across the literature as valuable components of student learning [10,15,29,41,42], suggesting these approaches may warrant greater integration within educational programs [10].

Other commonly reported educational strategies included blended learning approaches that enhanced flexibility and learner engagement [17,18,19], collaborative learning activities that fostered peer support and reciprocal learning [48], feedback-rich learning environments that strengthened self-assessment and competence development [23,24,25], and mentorship models that supported professional identity formation and transition to practice [32,44,47,53].

Curriculum mapping may provide one mechanism for integrating these strategies in a planned and progressive manner rather than isolated learning experiences, particularly given the fragmentation and variability reported across studies [7,35,54]. In addition, alignment with national accreditation standards (e.g., country-specific midwifery accreditation standards, ICM Global Standards for Midwifery Education, WHO Global Standards for Midwifery Education) may support greater consistency and accountability across programs [4,60].

The review highlights considerable variability in key learning experiences across programs, suggesting that future research and policy discussions may benefit from exploring whether greater consistency in simulation exposure, continuity-of-care opportunities, and reflective activities could support student preparedness [58]. Any future approaches aimed at increasing consistency would likely need to retain flexibility for local adaptation, such as differences between rural and metropolitan contexts, while potentially reducing unnecessary variability in student learning experiences [7].

Given the consistent reporting of transition shock and emotional burden across the reviewed studies, structured transition supports, peer networks, regular debriefing opportunities, and access to wellbeing resources emerged as potentially important components of future educational and transition models. These implications were derived from consistent reports of transition shock, emotional burden, inconsistent supervision, and the value of peer and mentor support across multiple studies [15,29,44,47,50]. Professional identity formation emerged throughout the literature as a developmental process that is supported by mentorship, reflective learning, high-quality clinical education, ethical guidance, and opportunities for progressive autonomy [8].

The findings suggest that educator, preceptor, and mentor capability may play an important role in the successful implementation of competency-based, reflective, and simulation-based educational approaches. Priority areas include facilitating reflection and debriefing, providing high-quality feedback, implementing simulation pedagogy, assessing higher-order competencies, and supporting students’ emotional wellbeing [9]. These priorities reflect recurring capability gaps identifies among educators, mentors, and supervisors throughout the reviewed literature [9,23,28,39,57]. Several included studies identified educator workload, preparation, and resource constraints as barriers to implementation, suggesting that educator capability warrants consideration within future educational planning.

### 4.2. Educational Framework for Midwifery Student Preparedness and Transition to Practice

Consistent with the aim of informing the development of a future educational framework, the synthesis identified a conceptual model for midwifery student preparedness and transition to practice comprising three interconnected domains: Approaches to Learning, Practical Learning, and Student Experience. Together, these domains provide a framework for understanding how educational, clinical, and developmental factors interact to influence preparedness for professional practice.

**Figure 3:**
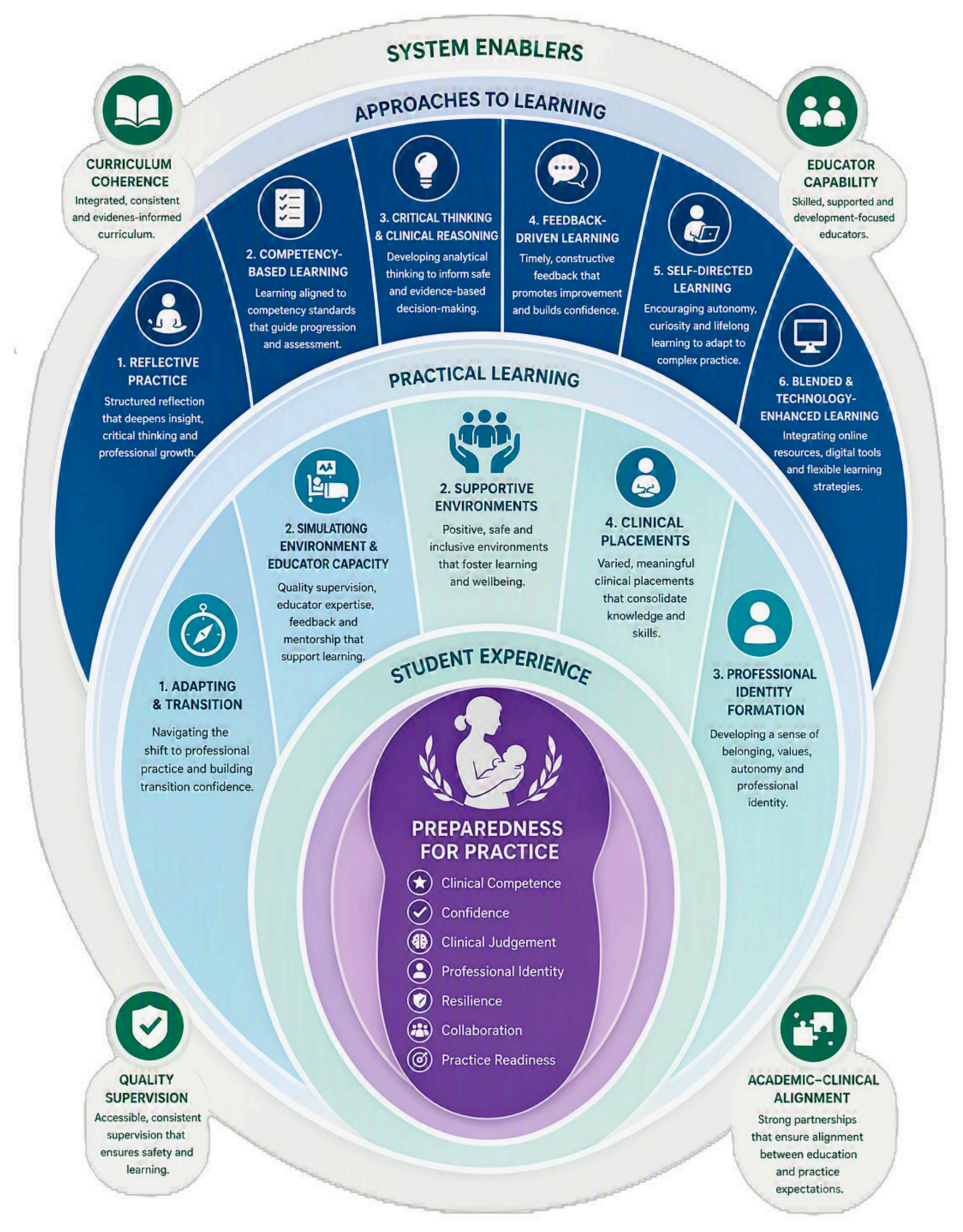
Educational framework for midwifery students.

Approaches to learning provide the educational foundation through reflective practice, competency-based learning, feedback processes, critical thinking, and self-directed learning. These approaches are operationalised within the practical learning domain through supportive learning environments, simulation, authentic clinical experiences, and collaborative learning opportunities. Together, these learning experiences shape the student’s experiences by fostering confidence, resilience, professional identity formation, adaptation to practice, and transition readiness. The framework therefore proposes that preparedness for practice develops through a dynamic interaction between pedagogical approaches, practice-based learning experiences, and developmental student outcomes rather than through any single educational strategy (Figure 3).

More specifically, reflective practice, learning environment and educator capacity, simulation and authentic practice, collaborative learning, adapting and transition, supportive environments, and professional identity formation represent interconnected sub-domains within the framework. Rather than operating independently, these components collectively contribute to preparedness for practice by supporting the development of competence, confidence, clinical judgement, resilience, and professional identity Their interdependence highlights the importance of viewing student preparedness as a dynamic and developmental process that extends across both educational and clinical contexts.

A key contribution of this framework is the integration of previously fragmented areas of inquiry into a single conceptual model. Rather than considering learning approaches, clinical experiences, or transition outcomes in isolation, the framework highlights how these elements interact to influence preparedness for practice. In doing so, it provides a coherent structure that may inform curriculum development, educator preparation, clinical learning design, and future research.

Across all domains, educator capability, curriculum coherence, supervision quality, and alignment between academic and clinical expectations appear to function as enabling factors. The findings suggest that preparedness for practice is not achieved through any single educational intervention but rather through the integration of these interconnected components. The proposed framework therefore provides a conceptual representation of how educational, clinical, and developmental factors may collectively support readiness for midwifery practice and offers a foundation for future empirical testing and refinement.

The model illustrates the interrelationship between approaches to learning, practical learning, and student experience in shaping preparedness for practice. System enablers, including curriculum coherence, educator capability, quality supervision, and academic–clinical alignment, support the development of competence, confidence, clinical judgement, professional identity, resilience, collaboration, and practice readiness among midwifery students.

### 4.3. Limitations

This scoping review offers a comprehensive overview of contemporary midwifery education by mapping evidence across approaches to learning, practical learning environments and student experience. A key strength of the review is the inclusion of literature from multiple countries, enabling consideration of diverse educational and practice contexts that influence midwifery preparation.

However, several limitations are acknowledged. Although studies were identified from a range of countries, their geographical distribution was uneven, with some regions contributing substantially more evidence than others. This imbalance may limit comparisons across educational systems and regulatory environments, particularly where midwifery education is influenced by region-specific standards and policies, for example, the common directives governing midwifery education within the European Union. Furthermore, the included studies were undertaken within diverse healthcare systems and models of maternity care, which may influence expectations of midwifery practice, professional scope, and educational preparation. Consequently, findings should be interpreted with recognition of these contextual differences.

Only articles published in English were included, which may restrict the applicability of the findings. Finally, consistent with scoping review methodology, the methodological quality of the included studies was not formally appraised, thus the synthesis reflects the strengths and weaknesses of the existing evidence base and therefore should be interpreted with caution.

## 5. Conclusions

Midwifery education is moving towards more student-centred, competency-based approaches; however, progress is uneven. Reflective practice, simulation, collaborative learning, and supportive clinical environments are consistently valued; however, implementation is inconsistent across programs. Higher-order capabilities such as critical thinking, clinical reasoning, professional identity, resilience and collaboration are often assumed to develop naturally, rather than being deliberately and systematically fostered. Fragmented innovation, variable alignment with competency frameworks and limited educator capacity further contribute to gaps in preparedness for practice. These findings highlight the need for integrated educational systems that operationalise the seven approaches to learning cohesively across curricula, learning environments, and supervision structures. Strengthening educator capability, curriculum coherence and supportive transitions into practice will be critical for preparing graduates who are confident, adaptable and equipped for the complexities of contemporary midwifery practice. No public involvement in any aspect of this research. This manuscript was drafted against the PRISMA Extension (PRISMA-ScR) [13] for Scoping Reviews. During the preparation of this work the author(s) used Microsoft Copilot (Version 2606, Build 20131.20152) in order to check for any grammatical or spelling issues. After using this tool/service, the author(s) reviewed and edited the content as needed and take full responsibility for the content of the publication.

## Figures and Tables

**Table 1:** Search terms.

Concept	Search Terms
Population	“Midwifery”[Mesh] OR “Midwife*”[Text Word] OR “Midwiv*”[Text Word] OR “Nurse Midwives”[Mesh] OR “Nurse Midwive*”[Text] OR “Nurse Midwife*”[Text] NOT “Traditional Birth Attendant*”, “Birth Attendant*”, “Tradition*”
Student Status	“student*” OR “Students, Health” OR “Occupations” OR “trainee*”
Career Stage	“early career”[Text Word] OR “ECM” OR “Begin*”[Text Word] OR “New”[Text Word] OR “newly”[Text Word] OR “graduate*”[Text Word] OR “junior”[Text Word] OR “probation*”[Text Word] OR “transition to practice”[Text Word]
Education	“Education”[Text Word] OR “Universities”[Text Word] OR “Professional Education”[Text Word] OR “Nursing Education, Research”[Mesh], OR “Nursing Schools”[Text Word] OR “Midwif* Education [Text Word] OR, “Midwif* Training”[Text Word], “Education, Training”[Mesh]
Competence and Readiness	“Professional Competence”[Mesh] OR “Self Efficacy”[Mesh] OR “Prepare*” OR “Ready” OR “readiness” OR “Competenc*”
Transition and Development	“Transition*”[Text Word], OR “Transition practice”[Text Word] OR “Gap” OR “Gaps” OR “professional development”

## Data Availability

No new data were created or analyzed in this study. Data sharing is not applicable to this article.

## Supplementary Materials

The following supporting information can be downloaded at: https://www.mdpi.com/article/10.3390/healthcare14152352/s1, Supplementary File S1 containing the PRISM-ScR checklist.

## Appendix A

**Table A1:** Literature review search results.

Database	Search String	Number of Results Loaded to EndNote
Web of Science	((((Midwifery) OR (“Nurse Midwives”)) OR (Midwife OR Midwiv* OR Nurse-Midwife OR Nurse-Midwives OR “Nurse Midwiv*” OR “Nurse Midwife*”)) AND (((Students) OR (“Students, Health” Occupations)) OR (student* OR Trainee* OR “early career” OR ECM OR “Early career midwif*” OR Begin* OR Newly OR Graduat* OR Junior OR Probation OR “Transition to Practice”)) AND ((((((Education) OR (Universities)) OR (Professional Education)) OR (“Nursing Education” Research)) OR (Nursing Schools)) OR (“Midwif* Education, “Midwif* Training”, Education, Training”)) AND (((Professional Competence) OR (Self efficacy)) OR (prepar* OR ready OR readiness OR competenc*)) AND (transition* OR “Transition practice” OR Gap OR Gaps OR “Professional development”))	520
Scopus	( ( ( INDEXTERMS ( midwifery ) ) OR ( INDEXTERMS ( “Nurse Midwives” ) ) ) OR ( TITLE-ABS-KEY ( midwife ) OR TITLE-ABS-KEY ( midwiv* ) OR TITLE-ABS-KEY ( nurse-midwife ) OR TITLE-ABS-KEY ( nurse-midwives ) OR TITLE-ABS-KEY ( “Nurse Midwiv*” ) OR TITLE-ABS-KEY ( “Nurse Midwife*” ) ) ) AND ( ( ( INDEXTERMS ( students ) ) OR ( “Students, Health” INDEXTERMS ( occupations ) ) ) OR ( TITLE-ABS-KEY ( student* ) OR TITLE-ABS-KEY ( trainee* ) OR TITLE-ABS-KEY ( “early career” ) OR TITLE-ABS-KEY ( ecm ) OR TITLE-ABS-KEY ( “Early career midwif*” ) OR TITLE-ABS-KEY ( begin* ) OR TITLE-ABS-KEY ( newly ) OR TITLE-ABS-KEY ( graduat* ) OR TITLE-ABS-KEY ( junior ) OR TITLE-ABS-KEY ( probation ) OR TITLE-ABS-KEY ( “Transition to Practice” ) ) ) AND ( ( ( ( ( ( INDEXTERMS ( education ) ) OR ( INDEXTERMS ( universities ) ) ) OR ( professional INDEXTERMS ( education ) ) ) OR ( “Nursing Education” INDEXTERMS ( research ) ) ) OR ( nursing INDEXTERMS ( schools ) ) ) OR ( TITLE-ABS-KEY ( “Midwif* Education, “ midwif* AND training “, Education, Training” ) ) ) AND ( ( ( professional INDEXTERMS ( competence ) ) OR ( self INDEXTERMS ( efficacy ) ) ) OR ( TITLE-ABS-KEY ( prepar* ) OR TITLE-ABS-KEY ( ready ) OR TITLE-ABS-KEY ( readiness ) OR TITLE-ABS-KEY ( competenc* ) ) ) AND ( TITLE-ABS-KEY ( transition* ) OR TITLE-ABS-KEY ( “Transition practice” ) OR TITLE-ABS-KEY ( gap ) OR TITLE-ABS-KEY ( gaps ) OR TITLE-ABS-KEY ( “Professional development” ) )	187
PubMed	(((Midwifery[MeSH Major Topic]) OR (“Nurse Midwives”[MeSH Major Topic])) OR (Midwife[Text Word] OR Midwiv*[Text Word] OR Nurse-Midwife[Text Word] OR Nurse-Midwives[Text Word] OR “Nurse Midwiv*”[Text Word] OR “Nurse Midwife*”[Text Word])) AND (((Students[MeSH Major Topic]) OR (Students, Health Occupations[MeSH Major Topic])) OR (student*[Text Word] OR Trainee*[Text Word] OR “early career”[Text Word] OR “ECM”[Text Word] OR “Early career midwif*”[Text Word] OR “Begin*”[Text Word] OR “Newly”[Text Word] OR Graduat*[Text Word] OR Junior[Text Word] OR Probation[Text Word] OR “Transition to Practice”[Text Word])) AND ((((((Education[MeSH Major Topic]) OR (Universities[MeSH Major Topic])) OR (Professional Education[MeSH Major Topic])) OR (Nursing Education Research[MeSH Major Topic])) OR (Nursing Schools[MeSH Major Topic])) OR (“Midwif* Education”, “Midwif* Training”, Education, Training[Text Word])) AND (((Professional Competence[MeSH Major Topic]) OR (Self efficacy[MeSH Major Topic])) OR (prepar*[Text Word] OR ready[Text Word] OR readiness[Text Word] OR competenc*[Text Word])) AND (transition*[Text Word] OR “Transition practice”[Text Word] OR Gap[Text Word] OR Gaps[Text Word] OR “Professional development”[Text Word])	96
APA PsychInfo	SU (SU (Midwifery OR Midwife OR Midwiv* OR “Nurse Midwives”) OR AND SU (“Professional education” OR “Professional competence” OR “Self efficacy” OR “midwif* train*” OR “midwif* educat*”)) OR TI ((“student* midwif*” OR “midwif* Student*”)) OR AB (AB (“student* midwif*” OR “midwif* Student*”)) AND SU (“Professional competence” OR “Self efficacy”) AND SU (“Nursing Education Research” OR “Nursing Schools” OR “Professional Education”) OR AB (“midwif* train*” OR “midwif* educat*”) OR TI (“midwif* train*” OR “midwif* educat*”)	165
Informit	[Midwifery OR “Nurse Midwives” OR Midwife OR Midwiv* OR Nurse-Midwife OR Nurse-Midwives OR “Nurse Midwiv*” OR “Nurse Midwife*”] AND [“health occupations students” OR “Midwif* student*” OR Student* OR Trainee* OR “Early career” OR “ECM” OR “Early career Midwif*” OR Begin* OR Newly OR Graduat* OR Junior OR Probation OR “Transition to practice” OR “Professional education” OR “Nursing education research” OR “Nursing School*” OR “Universities” OR “Education” OR “Professional Competence” OR “Self efficacy” OR prepar* OR ready OR Readiness OR competenc* OR transition* OR “transition practice” OR Gap OR Gaps OR “professional development”] AND [ ‘professional education’ OR ‘nursing education research’ OR ‘nursing school*’ OR ‘universities’ OR ‘education’] AND [ ‘professional competence’ OR ‘self efficacy’ OR prepar* OR ready OR readiness OR competenc*] AND [ transition* OR ‘transition practice’ OR gap OR gaps OR ‘professional development’] AND Limit To: Full Text AND Publication Date: (1 January 2004 TO 31 December 2024)	33
CINAHL	(MH “Midwifery+” OR MH “Nurse Midwifery” OR TI (midwife* OR midwive*) OR AB (midwife* OR midwive*)) AND (MH “Students, Midwifery” OR TI (“student*” OR “trainee*” OR “early career” OR “ECM” OR Begin* OR New OR Newly OR graduate* OR Junior OR probation* OR Start* OR transition) OR AB (“student*” OR “trainee*” OR “early career” OR “ECM” OR Begin* OR New OR Newly OR graduate* OR Junior OR probation* OR Start* OR transition)) AND (MH “Education+” OR TI (education OR training) OR AB (education OR training)) AND (MH “Professional competence+” OR MH “Self-Efficacy” OR TI (Prepare* OR ready OR Readiness OR Competenc*) OR AB (Prepare* OR ready OR Readiness OR Competenc*)) AND (TI (transition OR Gap OR Gaps OR “professional development”) OR AB (transition OR Gap OR Gaps OR “professional development”))	135
ProQuest Central	(MAINSUBJECT.EXACT(“Midwifery”) OR title(midwife* OR midwive*) OR abstract(midwife* OR midwive*)) AND (MAINSUBJECT.EXACT(“Students”) OR title(“student*” OR “trainee*” OR “early career” OR “ECM” OR Begin* OR New OR Newly OR graduate* OR Junior OR probation* OR Start* OR transition) OR abstract(“student*” OR “trainee*” OR “early career” OR “ECM” OR Begin* OR New OR Newly OR graduate* OR Junior OR probation* OR Start* OR transition)) AND (MAINSUBJECT.EXACT(“Education”) OR title(education OR training) OR abstract(education OR training)) AND (MAINSUBJECT.EXACT(“Professional standards”) OR title(Prepare* OR ready OR Readiness OR Competenc*) OR abstract(Prepare* OR ready OR Readiness OR Competenc*)) AND (title(transition OR Gap OR Gaps OR “professional development”) OR abstract(transition OR Gap OR Gaps OR “professional development”))	85

## Appendix B

**Table A2:** Overview of identified articles.

Author (Year)	Country	Study Design	Sample	Outcomes	Education Implications	Future Implications
Ahmadi et al., (2018) [34]	Iran	A qualitative study using semi-structured interviews and focus groups	Eighteen midwifery students Tehran University of Medical Sciences (*n* = 7), Shahid Beheshti University of Medical Sciences (*n* = 5), and Isfahan University of Medical Sciences (*n* = 6)	Six broad themes: limited opportunities to experience skills difficulties with the course plan gaps need for creating a supportive clinical environment learning learning drives confusion between different methods stress in the clinical setting	Universities are required to harmonise the number of students they admit to the educational program with their clinical capacities in a way that ensures students’ access to learning opportunities. Reviewing the midwifery course curriculum to determine if it is possible to provide the obstetrics study units continuously and without time gaps. Universities should provide programs for expanding the clinical instructors’ competencies in terms of educational and clinical skills. The methods of performing procedures should be unified between different clinical trainers.	Future exploration is needed to determine whether it is viable to provide increased pre-clinical hours in skill labs to better prepare the students for the transition to clinical practice.
Amod (2022) [52]	South Africia	Quantitative, descriptive design using a self evaluation questionnaire.	60 4th year undergraduate midwifery students from a university in KwaZulu-Natal. 43 females, 17 males.	88.6% of students perceived themselves as competent in midwifery procedures. 93% found clinical support beneficial	11.4% reported incompetence in key areas such as pap smears, pelvic assessments, neonatal resuscitation, and episiotomy suturing. Gaps in clinical skills suggest a need for curriculum and placement. improvements. Extended placements in continuity of care models like MGP foster deeper learning, professional identity formation, and confidence.	Develop formal mentorship training programs for midwifery practitioners. Integrate targeted training for procedures where students report low competence. Further research needed to explore midwifery practitioners’ perspectives on student support.
Arias & Coxon (2018) [42]	UK	Reflective teaching practice	Small teaching groups of 20	The experience of simulation and skills training reinforces the theory with practical knowledge for students. The classroom setting offered students a supportive setting in which to experiment with different SSMP approaches with a view to personalising care and without compromising safety.	The experiential model offers a low-resource approach to teaching SSMP and has potential application in other countries.	Further research needs to be undertaken to consider how this session of SSMP has impacted the students’ practice, post-qualification. Longitudinal research is needed to examine whether the experiential model described in this paper has the potential to improve students’ confidence in undertaking SSMP after qualification.
Austin (2020) [28]	New Zealand	Mixed methods (online survey with quantitative and qualitative questions)	41 midwifery students (2nd and 3rd year)	Majority reported improved reflective skills, ability to identify strengths, gaps, and set goals; valued one-to-one educator feedback; noted inconsistencies in educator support and need for better scaffolding	Emphasize structured reflective tools integrated into curricula; provide clear guidance and consistent educator feedback; scaffold learning across program years	Strengthen educator training in reflective facilitation; refine PDT to maintain engagement; explore impact of assessment on reflective learning; ensure culturally responsive support
Baird et al., (2022) [61]	Australia	Qualitative descriptive study using focus groups and thematic analysis	15 3rd year Bachelor of Midwifery students. No datal of male/female participants.	Developed strong professional identities and confidence in providing holistic, woman-centred care. Students appreciated the continuity of care model and the supportive relationships with preceptors.	Preceptors play a critical role in student development and should be trained and supported.	Expand MGP and continuity of care models to accommodate more students and new graduates.
Baird et al., (2016) [16]	Australia	Evaluation of an e-portfolio capstone assessment item using a peer review process. Quantitative survey and qualitative thematic analysis.	Convenience sample of academics (*n* = 20) from midwifery, nursing, and education from four universities, predominantly Australian (*n* = 19) plus one international academic (USA).	Academics rated the quality of the assessment highly. The e-portfolio met nine quality indicators. It was viewed as an authentic, engaging assessment promoting ongoing professional development and lifelong learning. Reflection, SWOT analysis, and professional philosophy development were highly valuable for student transformation.	E-portfolio capstone assessment item within a Bachelor of Midwifery program. Incorporates reflection (using Bass Model), SWOT analysis, professional philosophy statement, and comparison of clinical outcomes with national statistics.	Further development of the marking rubric is necessary for consistency. Further research should assess the acceptability of the assessment within a midwifery student population. Early student engagement with the e-portfolio is recommended for technical familiarity.
Blaka (2006) [32]	Norway	A qualitative case study using participant observation and semi-structured interviews.	Seven first-year midwifery students and their midwifery preceptors	Three themes emerged: being welcomed and accepted; a supportive dialogue between the preceptor being in the right place at the right time.	Midwifery must be familiar with situational knowledge, a competence that could only be acquired through participating in this community of practice. Preceptors play a key ‘gatekeeper’ during the process of learning skilled practice in the maternity wards for students.	Reflecting on experience may help students develop situational knowledge and tacit knowledge essential for competent midwifery practice.
Bradshaw (2018) [29]	Ireland	Descriptive qualitative study using focus group	13 final year midwifery students across two clinical sites	Consolidation of skills and confidence	Internship consolidates theoretical and clinical skills, inconsistencies in documentation, equipment and expectations across sites hinder learning, continuity of supervision and opportunities are important for independent practice	Developed structured pre-internship placements, provide formal support for preceptors, balance academic and clinical workload
Broad et al., (2011) [38]	UK	Evaluation	Final-year nursing and midwifery students at Keele University. Two cohorts were evaluated post-implementation of the transition module.	Increased confidence and competence among students entering professional roles. Improved preparedness in areas such as: Medicines management Planning and prioritization of patient care Professional issues (e.g., NMC Code, confidentiality) Equipment safety and basic life support Positive feedback from students regarding the value of early exposure to preceptorship concepts.	Embedding preceptorship principles into undergraduate education can ease the transition to practice. Shared learning between academic and clinical staff enhances curriculum relevance and student support. Early identification of students needing additional support allows for tailored interventions.	Potential for earlier integration of transition content in the curriculum, as suggested by student feedback. Model may serve as a template for other institutions seeking to improve transition support. Supports succession planning and retention by fostering a culture of lifelong learning and professional development. Encourages strategic partnerships between universities and healthcare providers to enhance workforce readiness.
Carolan (2013) [49]	Australia	Qualitative study	Participants: 30 third-year Bachelor of Midwifery students (out of 31 invited) Location: Melbourne, Australia	Three major themes emerged: A Skilled Practitioner Emphasis on clinical competence, emergency preparedness, evidence-based practice, accountability, and scope of practice. Recognition of the importance of ongoing professional development.	Curriculum Design: Need to address the gap between idealised views and clinical realities. Support development of realistic expectations and resilience. Support Structures: Early and ongoing support for professional identity formation. Structured reflection and discussion groups to process clinical experiences.	Research Gaps: Limited exploration of how students reconcile ideal vs. actual practice. Need for longitudinal studies tracking transition into graduate roles. Practice Recommendations: Develop targeted support programs to reduce attrition and burnout. Foster realistic expectations and coping strategies for early career midwives. Encourage mentorship and role modelling in clinical placements.
Cassidy et al., (2020) [21]	UK	Discussion paper	Protocol development drew on findings from original grounded theory study of UK nurse assessors/practice educators. Protocol was developed and piloted by a national stakeholder group across agreed sites in Wales UK.	The Action Plan Protocol is suggested as a strategy to address the issue of “failing to fail”. It provides a structured format (Assessment Footprint, Action Plan Template, Debriefing Checklist) for feedback and decision credibility review. Piloting yielded positive feedback from staff.	The Action Plan Protocol is a strategy used when pre-registration nursing and midwifery students are not meeting required levels of proficiency in clinical practice. It prompts the documentation of specific, measurable, attainable, relevant, time-bound (SMART) student learning objectives.	The authors propose further discussion on the utility of such protocols in general.
Cummins et al., (2014) [50]	Australia	Action research	First year Bachelor of Midwifery (BMid) students. Focus groups (*n* = 16). Survey respondents (*n* = 44 out of 50; 88% response rate).	Students positively rated the workshop, vodcasts, and simulated handover in reducing culture shock. 78% of students found the workshop highly useful in preparing for clinical practice. Themes identified included feeling awkward, coping with unexpected outcomes of birth, and valuing support from senior students/clinical facilitators.	Preparatory workshop held prior to clinical placement. Resources included vodcasts (peer advice, clinical educators’ tips, simulated handover), a ‘treasure box’ of equipment, and terminology activities.	Recommended that complications of labour and birth be included in the first semester curriculum due to unexpected exposure. Future studies should examine the impact of improved preparation on culture shock levels in other cohorts.
Ekelin (2016) [9]	Sweden	Qualitative context analysis	388 reflections from 18 midwifery students across six hospitals	Development from fragmented to holistic reflections	Daily reflective writing supports students’ transition to midwifery competence, reflections help integrate theory with practice and foster emotional and professional growth, supervisor feedback enhances learning but varies in quality affecting pedagogical value	Train supervisors to provide meaningful and structured feedback, use reflections to identify students’ learning needs and support development, avoid grading reflections to maintain honesty and depth in student writing
Embo et al., (2010) [25]	Belgium	Qualitative focus group study.	Four focus group discussions with second (*n* = 23) and last year (*n* = 10) Midwifery students (*n* = 33).	Integrating feedback and assessment (MAFI) supports active involvement in learning (collecting, writing, reading feedback). This process stimulates reflection and strategy development for improvement. MAFI supports self-assessment and formative assessment, but its value for summative assessment is disputed.	The Midwifery Assessment and Feedback Instrument (MAFI) integrates competency-based feedback and assessment to foster self-directed learning (SDL) in clinical practice. Students are responsible for taking the initiative to ask for feedback and reflect on competencies.	Future research should address the value of MAFI on summative assessment. Implementation requires training for students and supervisors, and dedicated time for reflective writing and dialogue.
Fasan et al., (2012) [22]	Italy	Four-stage, multi-method study	Midwives (*n* = 108 answered) working in 11 regional facilities. Graduands (*n* = 24) from two regional universities.	The average perceived independence score among graduands was 717.4 (out of 1020). 20.8% (5 students) had a perceived level of independence below sufficiency (cut-off 612 points). Highest independence perceived in trans-cultural care (average 8.1) and managing low-risk birth (average 7.7). Many students postpone graduation to gain more practical experience.	Direct entry midwifery bachelor degree (3 years duration). Focus on defining core competencies expected at registration to align academic and clinical expectations.	Curriculum changes should carefully consider the viewpoint of experts to protect and develop competence. Educational curricula may need rethinking due to the limited duration of the bachelor’s course (3 years, 5400 h).
Fenwick (2016) [45]	Australia	Descriptive qualitative study	11 students aged 20 years or less on enrolment into a Bachelor of Midwifery, 1st year (*n* = 1), 2nd Year (*n* = 4), 3rd year (*n* = 5) and a newly qualified midwife (*n* = 1).	Three themes: The Challenges of Being a Young Student Midwife. Finding your way. Making the transition from teenager to midwife	Taking a proactive stance in supporting young student’s understanding of the educational requirements.	Addressing poor professional behaviour in the workplace to ensure our younger students are adequately nurtured and supported. Focusing on the long term workforce outcomes for this group of students is also essential. Finding ways to gain opportunities of employment supports their learning and facilities emersion, as well as reducing financial burden. Developing and supporting students’ commitment, capability, purpose, and addressing issues related to poor interpersonal professional behaviour.
Geranmayeh et al., (2020) [23]	Iran	Quasi-experimental study.	120 fourth-year midwifery students (40 control, 40 verbal feedback, 40 written feedback).	Feedback significantly improved students’ self-assessment ability. Written feedback produced stronger correlation between instructor assessment and self-assessment (r = 0.74) compared to verbal (r = 0.63) or control (r = 0.38). Student–instructor agreement rates were highest in the written feedback group (77.5%).	Clinical midwifery education. Intervention groups received verbal or written feedback using the Sandwich Feedback Method (strengths, areas for revision, improvement interventions).	Educational planners should conduct programs for trainers to promote instructor abilities in giving constructive feedback and promote learners’ skills in self-guidance through self-evaluation. Future studies should use other methods and be more comprehensive.
Hajiesmaello (2022) [46]	Iran	Descriptive qualitative study	Midwifery students (*n* = 9), midwifery instructors (*n* = 6) and clinical midwives (*n* = 7).	Two themes: Discriminatory approach in the health system. Professional nature of midwifery.	Employed capable instructors who are interested in the profession and facilitate their active presence and participation in clinical decision-making.	Laying plans to modify the unfair structure of the systems, promote justice in existing clinical educational environments, expedite inter-professional cooperation and create learning opportunities for midwifery students in dedicated environments.
Holland et al., (2010) [51]	UK	Mixed-methods national evaluation	78 students, 78 mentors, 24 facilitators, 59 academics, 46 managers, 10 service users	Students found competent in skills but lacking in confidence and real-world readiness	Pre-registration programs must balance theory and real-life exposure; confidence-building needed	Formalised mentorship and guided post-reg support critical for student transitions
Hunter (2006) [33]	US	Qualitative descriptive study	Students in nurse-midwifery education program at San Diego State University and the University of California	Overwhelming support for the inclusion of storytelling into a weekly didactic midwifery course. Storytelling increased cognitive learning, enhanced role transition, and emotional clarification.	Nurse midwifery educators should consider incorporating this technique into education.	The benefits of storytelling strategy should be examined through more formal research study.
Janes et al., (2023) [18]	International (16 countries, e.g., UK, Canada, Hong Kong, Kenya)	Cross-sectional, sequential, mixed methods exploratory study	Nursing/nurse education leaders (*n* = 32) from across International Council of Nurses regions. Three follow-up case studies.	Benefits reported include flexibility, cost-effectiveness (mixed views), increased communication/interaction, and promotion of independent learning. Challenges included ensuring access to equipment/connectivity and staff/student preparation/support.	Blended learning (combined online and classroom-based). Methods included integrated online learning (75%), fully blended programs (15.6%), “flipped classroom,” videos, Moodle, and discussion boards.	Further work is needed on adequate preparation/support for staff/students, ensuring access to appropriate equipment and connectivity, addressing student perceptions that online learning is of lesser value, and comprehensive multi-stakeholder evaluation.
Jeffries et al., (2022) [35]	Australia	Qualitative descriptive study	Midwifery academics from several Australian universities	Identified rapid innovation in online learning, simulation, and inter-institutional collaboration to sustain program integrity.	Highlights the need for flexible, technology-supported learning and strong academic–clinical partnerships.	Recommend embedding digital strategies and preparedness planning into future curricula.
Kitson-Reynolds et al., (2015) [47]	UK	Case study and educational evaluation	Final-year student midwives and newly qualified midwives from NHS trusts	Improved transition experience, early clinical skill adoption, enhanced confidence	Final-year students benefit from preceptorship-like training before graduation	Widen adoption of transition passports; integrate induction and practice-based tasks pre-graduation Establish national consistency in preceptorship frameworks across NHS Trusts to ensure all newly qualified midwives receive equitable, structured support during their transition year.
Lake & McInnes (2012) [26]	UK	Action Research	Undergraduate Midwifery Programme documentation from one HEI. Focus group discussion with 10 student midwives (second and third year).	Findings indicated a lack of explicit emphasis on cognitive skill development in the curriculum. Students felt these skills developed “naturally” through clinical exposure and observed practice. Assessment primarily focused on practical skills and knowledge, not thinking skills.	Undergraduate midwifery curriculum utilizing Enquiry Based Learning (EBL) and simulation. Clinical learning heavily influenced by mentor role-modelling.	Change required in approach to clinical learning. Recommendations include incorporating specific cognitive theory content, redressing the balance of skills in practice profiles, and ensuring structured reflection/feedback in assessments.
Licqurish & Seibold (2013) [40]	Australia	Qualitative phenomenological study	Undergraduate midwifery students	Students struggled to achieve required numbers, felt tension between meeting quotas and providing woman-centred care.	Indicates need for realistic placement expectations, mentorship, and workload balance in programs.	Recommend policy and curriculum review to align competency standards with clinical placement realities.
Longworth (2013) [36]	UK	Mixed method	Thirty-three midwifery students at one university completed questionnaires (15/1st year, 11/2nd year, 7/3rd year, midwifery. Semi-structured interviews— 6 student midwives (purposively selected) from all year groups	Most midwifery students had positive views to learning and practising skills in the skills laboratory which led to improved confidence in performing skills on women and babies.	Identification of adequate instruction and appropriate designation of training space was important. However unrealistic mannequins and equipment hindered learning. Performing clinical skills in clinical practice provide a more enhanced learning experience	To improve women and babies care educators and mentors need to provide support and feedback that promotes the transfer knowledge and skill from the skills laboratory to the clinical environment.
McIntosh (2013) [54]	UK	Descriptive qualitative study	120 senior midwifery students from both three year and shortened program (18 months for those with registered nurse quantification)	Four themes: Teach yourself midwifery. Knowing it all. Right way of doing things. The importance of physical skills.	Involving students more deeply in curriculum design. Encourage midwifery educators to express the unknown-ness of midwifery practice and work with students to accept empirical knowledge as finite and mutable.	Bridge the divide in regard to cultures concerning the application of knowledge between regulatory bodies and university education.
Meegan & Martin (2020) [43]	UK	Qualitative design	Senior student midwives. Does not state numbers of completed interviews or where the students came from	Three themes identified—learning by doing, mentorship and transition to qualification.	The benefits of exposure to NIPE	Standardisation NIPR education and clinical practice required. Effective preceptorship programs to support newly qualified midwives to undertake NIPE. Educational facilities support to midwives providing mentorship to student midwives requires further development. Research required to examine the maintenance of the NIPE role post qualification.
Moloney et al., (2022) [41]	Ireland	Quantitative cross sectional design	67 respondents out of a possible 95 responded. Purposeful non-random convenience sample across four programmes at one university- BSc Nursing (Intellectual Disability), BSc Nursing (Mental Health), BSc Nursing (General) and BSc Midwifery	Simulation-based learning experiences provide important opportunities for students to build on previous learning, increase confidence and gain experience in preparation for real-life practice. It supports the development of applying critical thinking to a specific clinical scenario which in turn supports critically analysing their actions and decision-making.	Pre-briefing and debriefing are important aspects of the simulation that assists with increased confidence and supports meaningful learning. Time, cost and attention to design and implementation of effective simulation-based education strategies	Promote standards in simulation-based education strategies. To deliver effective simulation-based education programmes a cohesive approach needs to be employed—financial, resources and education all need to be considered.
O’Connor et al., (2018) [17]	Ireland	Mixed methods approach	Students (*n* = 50 responded to survey) and 20 stakeholders (7 students, 13 non-student stakeholders, including 2 midwives) who participated in the program delivered between 2013 and 2015.	Students positively evaluated blended learning and tutor support, finding the program relevant to practice and contributing to professional development/lifelong learning. Direct impact on clinical practice or health service delivery was difficult to ascertain.	Two-year Graduate Nurse/Midwife Programme (Postgraduate Certificate). Utilized a blended learning approach (face-to-face and online VLE). Support included dedicated tutors, recorded lectures, and synchronous/asynchronous chat rooms.	Development of future early graduate education programs is supported. Need for a clearer mechanism and more nuanced evaluation strategies to gauge the impact on practice.
Phillips & Hayes (2008) [27]	Australia	Theoretical paper	Graduate Diploma of Midwifery students (contextual focus, not empirical sample)	Positioning theory provides a framework for understanding identity formation and learning through conversations	Encourages integration of reflective positioning and professional conversations into midwifery curricula	Future research should examine structured use of positioning theory in practice settings and its impact on identity formation and learning
Power & Cole (2017) [19]	UK	Descriptive/Innovative Practice Report	Midwifery students at University of Northampton (no specific number reported)	Positive student feedback; increased confidence and preparedness; enhanced autonomy and adaptability	Active blended learning using video-assisted technology and simulation; modified four-stage teaching approach	Supports multidimensional learning; promotes digital fluency; aligns with evolving standards and complex clinical environments
Rance & Sweet (2016) [48]	Australia	Design based research	3 focus groups and one tutorial with peer education session. 14 3rd year and 60 1st year Bachelor of Midwifery students	The ability to co-create a culture for learning Reciprocal teaching and learning Development of clinical teaching capacity for students Teaching ability a ‘positive side effect’ of their midwifery training Students made explicit connections of the relational interdependence of: workplace-based experiences and their learning; workplace culture and clinical teaching; and the reciprocity of teaching and learning.	Revisit the intended, delivered and hidden curricula, and make explicit the intended learning outcomes to meet the competencies for practice. Understand the importance of teaching students how to teach	Similar research across multiple teaching facilities.
Shikuku (2024) [39]	Kenya	Qualitative study	20 nursing/midwifery training centres, looking at educators’ perspectives of a new training intervention, including mentoring and peer support for 12 months. 12 educators with intervention, 8 control educators, and 8 mentors participated.	Positive reaction to new content for educators and students, with mentoring/support building skills and confidence in educators, using contemporary knowledge. There was increased participatory and team/peer teaching practices, and improved feedback mechanisms between educators and students. Difficulties with limited time and infrastructure requirements.	A need to ensure educators have access to up to date resources, knowledge, skills, and continuing professional development	Future research on return on investments is needed.
Sidebotham (2015) [62]	Australia	Descriptive exploratory qualitative design	56 s- and third-year midwifery student for survey and 16 students for focus group	Early clinical exposure and continuity of care experiences significantly enhanced midwifery students’ sense of capability, identity, purpose, resourcefulness, and connectedness, contributing to their confidence, motivation, and retention in the program. Embedding woman-centred care philosophy and fostering supportive relationships with peers, lecturers, and clinicians were key enablers of student success.	Program delivery methods and student support systems should be designed to enable maximum flexibility to promote capability and resourcefulness and embed sense of purpose and identity early in the program.	The Five-Senses of Success framework could be contextually applied within any international setting to support students to achieve success in their degree program
Sidebotham et al., (2018) [15]	Australia	Mixed Methods	34 final-year Bachelor of Midwifery students; 30 completed survey; 29 participated in focus group	Increased confidence, critical thinking, professional identity, and preparedness for practice	Capstone e-portfolio assessment aligned with NMBA competencies; reflective practice; continuity of care analysis; personal development planning	Promotes lifelong learning and professional identity; recommended for broader implementation and further research
Sun (2022) [53]	China	Descriptive phenomenological study	fourteen final-year midwifery students who were undertaking clinical placemen	Two theme clusters: 1. positive experiences motivating professional identity development and 2. negative experiences impeding professional identity development Seven themes: feeling the sense of accomplishment for facilitating smooth births, developing professional competence, positive role models of clinical mentors, cooperative inter-professional relationships, high-intensity working state, emotional instability of birthing women, and feeling insufficient in professional competence	Adequate preparation prior to clinical placement, enhancement of professional competence, positive role models of clinical mentors, interprofessional collaboration, strengthening of the sense of accomplishment, learning of being with women, and alleviation of midwifery workforce shortage might contribute to midwifery students’ professional identity development during clinical placement and are therefore recommended.	Midwifery students’ professional identity development during clinical placement and provide a reference for midwifery educators and administrators so as to prepare confident and motivated midwives to provide high-quality maternal care and address the shortage of midwifery workforce.
Thompson et al., (2021) [24]	Netherlands	Quasi-experimental with mixed methods	Two cohorts of third-year midwifery students (Control: 65; Intervention: 47)	Significant increase in general and midwifery-specific self-efficacy; improved advocacy and communication skills	ESSENTIAL programme: workshops, debate training, Optimality Index use, persuasive communication strategies	Suggests self-efficacy training enhances advocacy; recommends development of validated midwifery self-efficacy scales and broader implementation
Vasilevski (2022) [44]	Australia	A qualitative study	21 first-year Bachelor of Midwifery students.	students reported varied experiences in the quality of education and support obtained on clinical placement with many students emphasised a need for both practical and theoretical learning experiences on placement, and that peer-mentoring had the potential to address these limitations and knowledge gaps.	Peer-mentoring that is designed to meet student needs is likely to enhance engagement, and thus improve academic outcomes, promote clinical competence, and reduce university attrition rates.	Appropriately designed clinical placement peer-mentoring programs may empower students to manage academic demands and adjust to the challenging hospital setting.
Yigzaw et al., (2015) [20]	Ethiopia	Cross-sectional study with mixed methods	484 graduating students from 25 public midwifery training institutions (TVET colleges and universities)	Mean competence score: 51.8%; only 31.6% met national passing standard; low scores in emergency obstetric skills; male gender, sufficient clinical experience, and number of births attended predicted higher competence	Competency-based and subject-based curricula; limited clinical exposure; OSCE used for assessment; students reported poor learning environments and inadequate supervision	Strengthen quality assurance in midwifery education; improve clinical training and supervision; implement in-service mentoring; consider accreditation for public institutions
Young (2012) [37]	UK	Ethnography	36 mid students 5 midwives <1 yr 12 midwifery mentors	Students learn through: Participation in daily practice as a midwife, copying and adapting, asking trusted colleagues, feel vulnerable with authoritarian behaviours impacting ability	Creating safe environments assists ability to learn and make decisions. Diverse decision making situations and pressures practiced.	The social and cultural aspects of the environment influence decision making ability
